# EEG-based emotion recognition using graph convolutional neural network with dual attention mechanism

**DOI:** 10.3389/fncom.2024.1416494

**Published:** 2024-07-19

**Authors:** Wei Chen, Yuan Liao, Rui Dai, Yuanlin Dong, Liya Huang

**Affiliations:** College of Electronic and Optical Engineering & College of Flexible Electronics (Future Technology), Nanjing University of Posts and Telecommunications, Nanjing, China

**Keywords:** attention mechanism, EEG, electrode channels, emotion recognition, frequency bands, graph convolutional neural network, transformer

## Abstract

EEG-based emotion recognition is becoming crucial in brain-computer interfaces (BCI). Currently, most researches focus on improving accuracy, while neglecting further research on the interpretability of models, we are committed to analyzing the impact of different brain regions and signal frequency bands on emotion generation based on graph structure. Therefore, this paper proposes a method named Dual Attention Mechanism Graph Convolutional Neural Network (DAMGCN). Specifically, we utilize graph convolutional neural networks to model the brain network as a graph to extract representative spatial features. Furthermore, we employ the self-attention mechanism of the Transformer model which allocates more electrode channel weights and signal frequency band weights to important brain regions and frequency bands. The visualization of attention mechanism clearly demonstrates the weight allocation learned by DAMGCN. During the performance evaluation of our model on the DEAP, SEED, and SEED-IV datasets, we achieved the best results on the SEED dataset, showing subject-dependent experiments’ accuracy of 99.42% and subject-independent experiments’ accuracy of 73.21%. The results are demonstrably superior to the accuracies of most existing models in the realm of EEG-based emotion recognition.

## 1 Introduction

EMOTION is the subjective emotional response that humans experience in specific moments or situations. It plays a vital role in correctly interpreting behavior and facilitating effective communication (Jenke et al., 2014). In the development of brain-computer interface (BCI) systems, the urgency to empower machines with the ability to assist in analyzing human emotions is significant (Gu et al., 2023). Consequently, emotion recognition has emerged as one of the crucial research directions in affective computing (Hu et al., 2019). Through numerous studies, it has been discovered that the generation of human emotions is highly correlated with electrical signals in the cerebral cortex of the brain (She et al., 2023). Additionally, humans may involuntarily or intentionally conceal their real emotions through facial expressions and language except EEG signals (Zhang et al., 2020). As a result, researchers prefer emotion recognition methods based on EEG signals as they are more reliable and objective in capturing an individual’s emotional state (Lu et al., 2023).

In earlier studies on emotion recognition, traditional machine learning methods were predominantly relied upon, such as Support Vector Machines (SVM) (Kumar and Nataraj, 2019), which were extensively used due to their effectiveness in handling high-dimensional feature spaces and their ability to perform well with a limited amount of training data. However, as deep learning continues to progress, we are now witnessing a shift in the landscape. It has not only demonstrated significant performance in the field of computer vision (Yuan et al., 2018; Zhang and Zheng, 2022) and natural language processing (Lauriola and Aiolli, 2022), but has also gained widespread popularity in biomedical signal processing (Rahman et al., 2021). Initially, Wang et al. (2022) utilized convolutional neural network (CNN) to classify positive, neutral, and negative emotions. Building on the premise that CNN plays a vital role in emotion detection, Yang et al. (2018) designed a parallel convolutional recurrent neural network model for emotion recognition, which yielded promising results. To further investigate the temporal and spatial aspects of brain networks, Cui et al. (2022) employed a CNN-BiLSTM architecture to investigate the temporal complexity and spatial location of brain networks. At the same time, some researchers (Jia et al., 2021; Yang et al., 2024) recognized that the brain exhibits a complex graph structure in three-dimensional space, leading to investigations from spatial perspective. Liu et al. (2024) provide a comprehensive and systematic review of existing graph neural networks in EEG-based emotion recognition. For example, Liu et al. (2023) and Ding et al. (2022) effectively leveraged Graph Convolutional Neural Networks for the efficient feature extraction through both global information aggregation between brain regions and local information integration within brain regions under emotional states. This represents a promising start in EEG-based emotion recognition research, yet they did not proceed to further explore critical factors for classifying emotions. Liu et al. (2023) utilized transformer model for multimodal knowledge extraction, thereby enhancing recognition performance. Gong et al. (2023) designed an attention-based feature extraction and fusion module, which can selectively obtain key features based on their spatial and temporal significances. Guo et al. (2022) delved into understanding the dependence of emotion recognition building on the transformer model on each EEG channel and visualized the captured features. While they succeeded in extracting vital information from time segments or channels, their approach overlooked the foundational graph structure. This oversight led to the loss of significant information, consequently capping the potential of their model’s classification capability.

To address these challenges, our study introduces a brain decoding approach that primarily relies on graph convolutional neural network and attention mechanism of transformer. To be precise, we construct a three-dimensional spatial adjacency matrix and employ graph convolutional neural networks to aggregate information from multiple channels, extracting representative spatiotemporal features. Additionally, we utilize two attention mechanisms: electrode channel attention and signal frequency band attention. These mechanisms reveal the contributions of individual electrode channels to emotional responses in different brain regions and the relative impact of various frequency bands on emotions. By employing these attention mechanisms, we effectively leverage the information embedded in EEG signals, leading to improved overall decoding performance. The main contributions can be summarized as follows:

1. A graph convolutional neural network framework with a dual attention mechanism is proposed by combining GCN and Transformer. Experiments were conducted on DEAP, SEED, and SEED-IV datasets, covering binary, ternary, and quaternary classification tasks. The subject-dependent and subject-independent experimental results demonstrate that our model outperforms most existing models.
2. The graph convolution operation aggregates brain channel features based on the adjacency matrix composed of three-dimensional distances, effectively utilizing spatial information. After that, dual attention mechanism operates on both electrode channels and signal frequency bands, allocates weights and allows for better extraction of crucial information from temporal and spectral EEG data sequences.
3. After model training, we visually analyze the dual attention mechanism through model parameters, which can better analyze the roles of different electrode channels and signal frequency bands in emotion processing. It provides valuable insights for further research on emotion regulation and cognitive processes, offering important clues for exploring these domains.

The remaining sections of this paper are organized as follows: Section 2 outlines the related work, providing context and background for our study. Section 3 describes the methodology, including the development and implementation of our model. Section 4 details the experimental setup, dataset, evaluation metrics and presents the results. Finally, Section 5 concludes the paper with a discussion of the implications, limitations, and future directions for research in brain science.

## 2 Related work

In this section, we begin by showcasing several notable emotional recognition features for EEG signals. After that, we provide a concise overview of GCN and attention mechanism, fundamental components of the proposed model.

### 2.1 Emotional recognition features for EEG signals

In general, most EEG-based emotion recognition methods begin by extracting features from processed EEG signals. Subsequently, these extracted features are then employed as input for classification algorithms to achieve accurate classification of emotional states (Cai et al., 2021).

Zhang et al. (2020) has indicated that the EEG features employed in emotion recognition can be broadly classified into time-domain, frequency-domain and time-frequency domain features. Recent studies (García-Martínez et al., 2021; Yan et al., 2023) shows that the human brain functions as a nonlinear dynamic system, with EEG signals analyzable via nonlinear methods and feature extraction. Commonly used nonlinear features for EEG signals include differential entropy (DE) (Duan et al., 2013), permutation entropy (Nicolaou and Georgiou, 2012), discrete wavelet transform (DWT) (Chen et al., 2017), power spectral density (PSD) (Alam et al., 2020) and various other entropy measures. Among them, DE was initially proposed by Duan et al. (2013) and validated to be effective in the field of emotion recognition. As a result, DE has gained significant popularity as a widely used and effective feature extraction technique in the domain of EEG-based emotion recognition.

### 2.2 Graph convolutional neural network

Graph Convolutional Neural Network is a deep learning model specifically designed for graph data (Kipf and Welling, 2017). It extends the idea of convolutional operations to the graph domain and can encode graph structures and node features in a useful way for semi-supervised classification. GCN has shown excellent performance in tasks such as social networks (Zhong et al., 2020), machine fault diagnosis (Li et al., 2021), and recommendation system (He et al., 2020). Since the brain can be considered as a complex graph network, GCN is capable of effectively capturing both local and global information in brain networks. This enables it to enhance the performance of EEG signal analysis and facilitate research and applications in neuroscience and neurology. Song et al. (2020) first applied GCN for EEG emotion recognition, using a Dynamic Graph Convolutional Neural Network (DGCNN) that operates on multi-channel EEG data. Qiu et al. (2023) introduced multi head attention mechanism and residual network, proposing the Multi-head Residual Graph Convolutional Neural Network (MRGCN) model which combines short-range and long-range connections for EEG-based emotion recognition.

### 2.3 Attention mechanism

Graph Attention Network (GAT) (Veličković et al., 2018), as a graph neural network based on the attention mechanism, has shown excellent performance in processing graph data. However, in brain network research, GAT has a limitation in capturing global information. The attention mechanism of GAT is based on the interaction between nodes for weighted aggregation, which may result in insufficient capture of global information in the entire brain network, especially in the presence of long-range dependencies. The Transformer model (Vaswani et al., 2023) can effectively address this limitation. The Transformer model initially caused a great sensation in natural language processing. Its attention mechanism can adaptively focus on different positions of information according to the task requirements. This adaptability enables the model to capture global relationships and better differentiate important information stored in multiple channels of EEG signals. As a result, many researchers have applied the Transformer model in EEG studies. Wang et al. (2022) utilized the Transformer encoder to capture spatial dependencies between brain regions. Liu et al. (2023) made full use of the relative spatial information in EEG data and constructed a dual-layer capsule network for emotion recognition. (Song et al. (2021) employed attention along the feature channel dimension to weight the preprocessed and spatially filtered data, while also considering the global dependencies along the temporal dimension for emotion recognition.

## 3 Method

**Figure 1:**
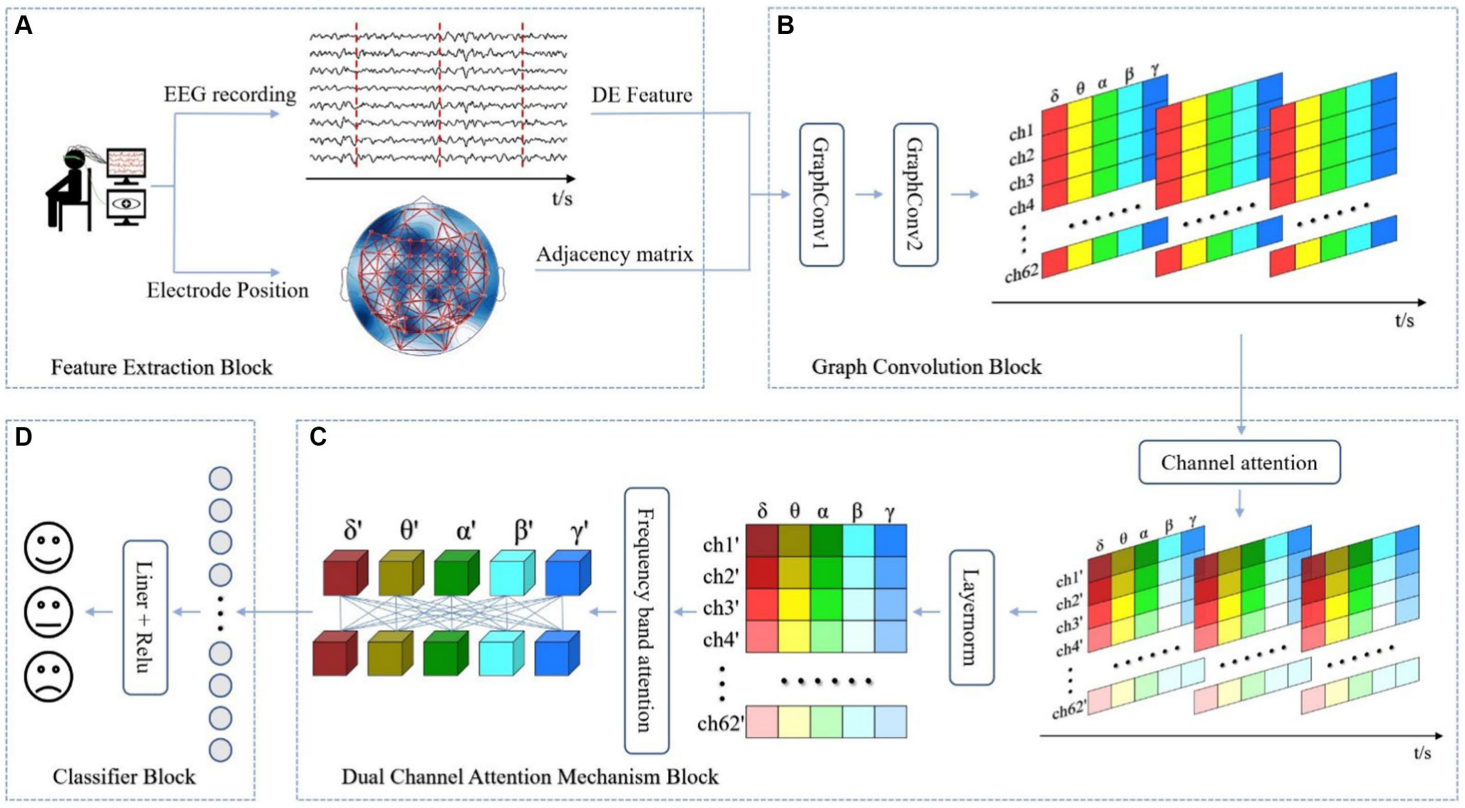
The overall framework of the proposed DAMGCN for Emotion Recognition. In **(A)** Feature Extraction Block, EEG signals are decomposed into five bands and adjacency matrix composed of three-dimensional electrode coordinate distances is established. **(B)** Graph Convolution Block utilizes the graph structure information to extract spatial topological features of the complex network. **(C)** Dual Attention Mechanism Block adaptively assigns weights to electrode channels and frequency band channels. Finally, the output results are obtained through **(D)** Classifier Block.

In section 3, we provide a comprehensive explanation of the DAMGCN model, as illustrated in Figure 1. The model comprises four main blocks: (A) Feature Extraction Block, (B) Graph Convolution Block, (C) Dual Attention Mechanism Block, and (D) Classifier Block.

### 3.1 Feature extraction block

As shown in Figure 1, we extract DE features from EEG signals and the three-dimensional spatial positions of electrodes for emotion classification. DE is a measure in information theory that describes the uncertainty of continuous random variables and defined as H(X) (Feutrill and Roughan, 2021):


(1) $$H\left(X\right)=-\overset{+\infty }{\underset{-\infty }{\int }}p\left(x\right)\log\left(p\left(x\right)\right)dx$$

where X is a time series, p(x) represents the probability density function of the continuous information. Assuming X as the EEG signal and following a Gaussian distribution $N\left(\mu ,\sigma^{2}\right)$ , μ and σ are the mean and variance of X , then p(x) can be expressed as shown in Eq. (2):


(2) $$p\left(x\right)=\frac{1}{\sqrt{2\pi \sigma^{2}}}e^{\left(-\frac{{\left(x-\mu \right)}^{2}}{2\sigma^{2}}\right)}$$

As a result, Eq. (1) can be expressed as:


(3) $$H\left(X\right)=\frac{1}{2}\log\left(2\pi e\sigma^{2}\right)$$

We divide the EEG signals into five frequency bands using the Short-Time Fourier Transform (STFT): δ wave (1–4 Hz), θ wave (4–8 Hz), α wave (8–13 Hz), β wave (13–30 Hz), and γ wave (>30 Hz). After that, we utilize the EEG data of the five wave bands as inputs to Eq. (3) to calculate DE.

After EEG signal processing, another feature that we need to extract is the adjacency matrix based on brain network nodes. 3D electrode coordinates are employed to compute the connectivity matrices of electrode channels to construct a three-dimensional spatial adjacency matrix, as shown in Eq. (4):


(4) $$\{\begin{array}{c} d_{ij}=\sqrt{{\left(x_{i}-x_{j}\right)}^{2}+{\left(y_{i}-y_{j}\right)}^{2}+{\left(z_{i}-z_{j}\right)}^{2}}\\ w_{ij}=\frac{1}{d_{ij}};\;\;if\;i\neq j\\ w_{ij}=1;\;\;if\;i=j \end{array}$$

This matrix describes the structure and connectivity patterns of the brain network, allowing us to analyze functional connections between nodes, study the complexity of the brain network. The adjacency matrix plays a crucial role in understanding the organization, functionality, and characteristics of the brain network (Gómez-Tapia and Longo, 2022).

### 3.2 Graph convolution block

**Figure 2:**
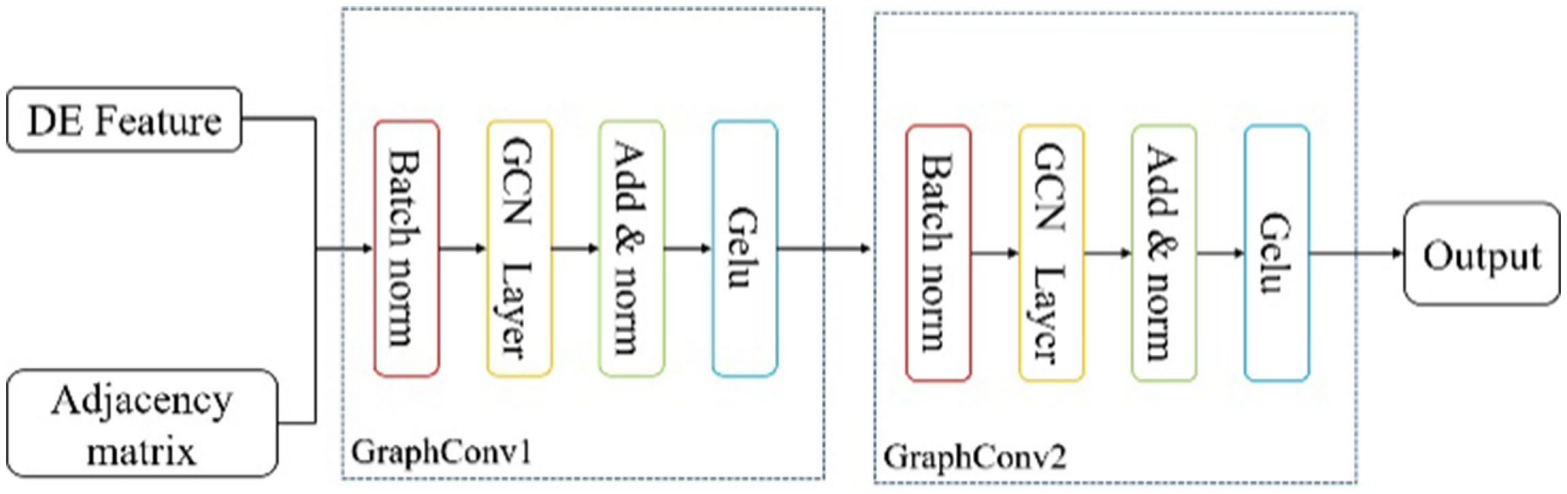
The specific implementation of Graph Convolution Block.

As shown in the Figure 2, this block consists of two graph convolution layers. The DE features and the adjacency matrix obtained from last block are input in batches into the Graph Convolution Block. In the GraphConv Model, the input is normalized using a batch normalization layer (Ioffe and Szegedy, 2015) to reduce the absolute differences between the data, thereby accelerating convergence speed and improving stability. In a batch of data $X^{B}=\left\{X_{1},X_{2},X_{3},\ldots ,X_{i}\right\}$ , $X_{i}\in R^{C\times F}$ , where $X^{B}$ has three dimensions called batch-size(B), channel(C), frequency(F) band, the normalization formula is as follows:


(5) $${\hat{y}}_{i}=\gamma \left(\frac{x_{i}-\mu }{\sqrt{\sigma^{2}+\epsilon }}\right)+\beta$$

$x_{i}$ denotes the dimension of channel, μ and σ represent the mean and standard deviation, ϵ is a small constant added to the batch variance for numerical stability, γ is the scaling factor, β is the shifting factor, and ${\hat{y}}_{i}$ represents the data after normalization.

The GCN layer convolves and aggregates the feature information of nodes using the adjacency matrix. The calculation formula can be concluded as follows (Kipf and Welling, 2017):


(6) $$H^{\left(l+1\right)}=\sigma \left({\tilde{D}}^{-\frac{1}{2}}\tilde{A}{\tilde{D}}^{-\frac{1}{2}}H^{\left(l\right)}W^{\left(l\right)}\right)$$

here, $H^{\left(l\right)}$ represents the node features at layer l. $W^{\left(l\right)}$ denotes the trainable weight matrix. $\tilde{A}=A+I_{N}$ is the self-connected adjacency matrix of the graph G, where A is the original adjacency matrix and $I_{N}$ is the identity matrix, ${\tilde{D}}_{ii}=\underset{j}{\sum }{\tilde{A}}_{ij}$ is the degree of matrix of $\tilde{A}$ . $\sigma \left(\cdot \right)$ is the activation function, typically a non-linear function like $\mathrm{ReLU}\left(\cdot \right)$ . We also cited the advantages of residual networks (He et al., 2015), including their facilitation of model training, alleviation of overfitting, and increased network depth. Thus, Eq. (6) can be further expressed as shown in Eq. (7):


(7) $$H_{res}^{\left(l+1\right)}=H^{\left(l+1\right)}+H^{\left(l\right)}$$

After passing through the residual network, the data is activated using the GELU activation function (Hendrycks and Gimpel, 2023), which is defined by Eq. (8):


(8) $$\mathrm{GELU}\left(x\right)=x*\sigma \left(1.702x\right)=x*\frac{1}{1+e^{-1.702x}}$$

### 3.3 Dual channel attention mechanism block

The dual attention mechanism block employs the attention mechanism of Transformer to separately allocate attention weights to the EEG channels and the frequency bands of the DE data. In order to fully utilize the graph structure information obtained by the graph convolution block, we first implement the electrode channel mechanism to enhance the emotional relevance of certain electrodes within the channels while suppressing the irrelevant ones. Frequency adaptation mechanism amplifies the impact of relevant frequency band signals on emotions while attenuating the influence of irrelevant frequency band signals.

**Figure 3:**
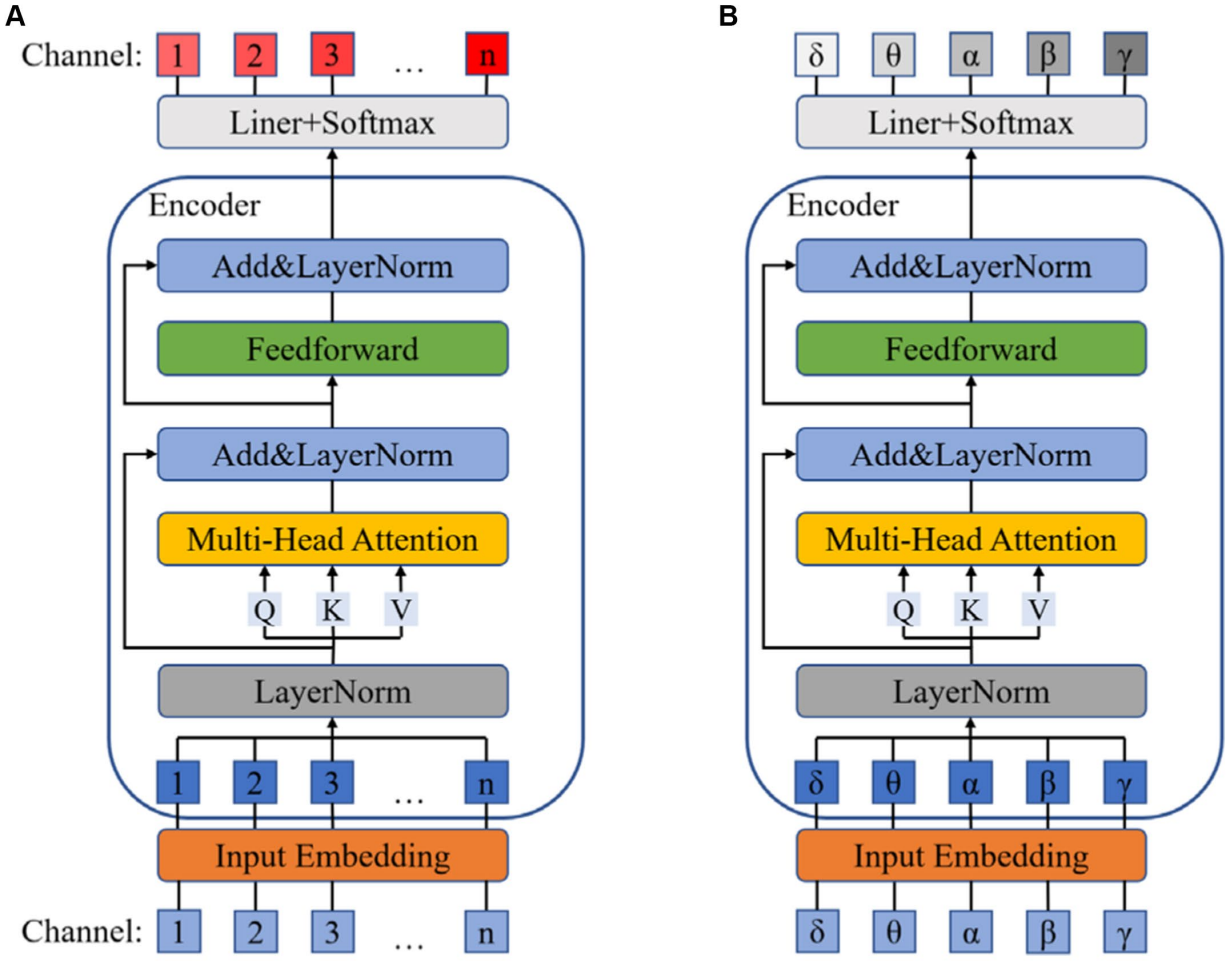
The structure of electrode channel adaptation **(A)** and frequency band adaptation **(B)**.

As shown in the Figure 3, the data after Graph Convolution Block is first mapped to a high-dimensional embedding vector $Y^{B}$ ( $Y^{B}=\left\{Y_{1},Y_{2},Y_{3},\ldots ,Y_{i}\right\},Y_{i}\in R^{C\times E}$ , where $Y^{B}$ has three dimensions called batch-size, channel, embedding-vector) through Input embedding and then normalized using LayerNorm (Ba et al., 2016). The formula is the same as Eq. (5), while $x_{i}$ represents the dimension of embedding vector here. Next, we transform the embedding vector into Query, Keys, and Values vectors using three weight matrices $W^{Q}$ , $W^{K}$ , and $W^{V}$ to compute the attention, as shown in Eq. (9):


(9) $$\left\{\begin{array}{c} Q=X\cdot W^{Q}\\ K=X\cdot W^{K}\\ V=X\cdot W^{V}\\ Attention=softmax\left(\frac{Q\cdot K^{T}}{\sqrt{d_{k}}}\right) \end{array}\right.$$

To enhance the robustness and stability of the model, we employ Multi-Head Attention to obtain multiple sets of Query, Keys, and Values. Each set is used to calculate a Z matrix separately, and the resulting Z matrices can be concatenated together, as shown in Eq. (10):


(10) $$Z=concat\left\{softmax\left(\left(\frac{Q_{i}\cdot K_{i}^{T}}{\sqrt{d_{k}}}\right)\cdot V_{i}\right),\;i=1,2,3\ldots \right\}$$

During the computation of the attention mechanism, we also employ residual connections and LayerNorm to prevent training degradation and other issues. Finally, the output is obtained through the forward propagation network based on residual connections, as shown in Eq. (11):


(11) $$output=\left(Z\cdot W^{T}+b\right)+Z$$

The frequency band attention mechanism is similar to the channel attention mechanism, and the flowchart is shown in the Figure 3.

### 3.4 Classifier block

During the model training phase, the feature vector obtained from the forward propagation is passed through a fully connected layer to achieve dimensionality reduction. This is followed by generating predicted labels, resulting in the final classification results. The cross-entropy loss function is then employed to calculate the loss between the true emotion labels y and the predicted emotion $\hat{y}$ labels, as shown in Eq. (12):


(12) $$\{\begin{array}{c} \hat{y}=argmax\left(output\right)\\ Loss\left(\theta \right)=-\overset{N}{\underset{i=1}{\sum }}y_{i}\log{\hat{y}}_{i} \end{array}$$

where θ represents all the parameters in the DAMGCN model. To evaluate the classification results of the DAMGCN model, we use accuracy as performance metrics, as shown in Eq. (13):


(13) $$Accuracy=\frac{\left(TP+TN\right)}{\left(TP+TN+FP+FN\right)}$$

The formula is an example of binary tasks. Total samples are the sum of true positive (TP) predictions, true negative (TN) predictions, false positive (FP) predictions, false negative (FN) predictions, with the sum of TP and TN representing the count of samples predicted correctly.

## 4 Experiment setting and results

In this section, we first introduce three different types of datasets and describe the experimental setup and preprocessing steps. Based on this, we mainly conduct subject-dependent experiments to demonstrate the EEG emotion classification performance of the proposed DAMGCN model and promote it in subject-independent experiments. Subsequently, we visualize the experimental results through subject-dependent experimental data for mechanism analysis and conducted ablation experiments.

### 4.1 Datasets

The DEAP dataset (Koelstra et al., 2012) collected physiological signals and emotion label data from 32 participants. Each participant watched 40 segments of audio-visual stimuli. EEG signals were captured using a 40-electrode EEG cap distributed according to the 10–20 system. The duration of each trial was 63 s, consisting of a 3-s baseline data at the start followed by 60 s of test data. The data was downsampled to 128 Hz and bandpass frequency filtering was applied in the range of 4.0–45.0 Hz. The labels were provided through questionnaire surveys to assess the emotional evaluation of the stimulus videos. The participants were instructed to provide subjective ratings for the stimulus videos across four dimensions: valence, arousal, dominance, and liking. The points ranged from 1 to 9 to express self-states, so we compromise by selecting a threshold of 5 to binarize the labels.

The SEED dataset (Zheng and Bao-Liang, 2015) contains EEG signal data from 15 subjects collected using the 62-channel ESI NeuroScan System. The database comprises three sessions, and within each session, participants were instructed to choose 15 segments for emotion elicitation. The data was downsampled to 200 Hz, and a bandpass frequency filter ranging from 0 to 75.0 Hz was applied. The emotion labels include three emotional states: positive, neutral, and negative.

The SEED-IV dataset (Zheng et al., 2019) is an extension of the SEED dataset, with the main difference being the videos viewed by the participants. Each session in SEED-IV consists of 24 trials, and the emotion classification labels are categorized into four classes: happy, sad, neutral, and fear.

In summary, the similarities and differences among the three datasets are shown in the Table 1. It should be noted that the duration of trials in the SEED and SEED-IV dataset are inconsistent.

**Table 1:** Datasets introduction.

Dataset	DEAP	SEED	SEED-IV
Channels	40	62	62
Sampling rate	128 Hz	200 Hz	200 Hz
Subjects	32	15	15
Sessions	1	3	3
Trials	40	15	72
Trial duration	63 s	120 ~ 240 s	120 ~ 240 s
Frequency range	4.0 ~ 45.0 Hz	0 ~ 75.0 Hz	1.0 ~ 75.0 Hz
Frequency bands	4	5	5
Label types	4	1	1
Classification	2	3	4

### 4.2 Data preprocessing

The approach to data preprocessing and feature extraction in emotion recognition tasks is critical for optimal model performance. Our methodology for processing the DEAP, SEED, and SEED-IV datasets is outlined as follows:

For the DEAP dataset, we selected 32 channels related to EEG and set the non-overlapping duration of each segment to 0.5 s to obtain 120 samples every trial. Since the provided data from the official source has already been filtered using a bandpass filter in the range of 4.0–45.0 Hz, according to the section 3.1 mentioned, we further filtered the raw data into four frequency bands (θ, α, β, γ) and extracted DE features. Data format for each subject is 4800×32×4(sample×channel×frequencyband) .

For the SEED and SEED-IV datasets, EEG data from each channel is segmented into temporal window of 1 s each, with no overlap between them. Unlike the DEAP dataset, SEED and SEED-IV do not filter out signals in the δ (1–4 Hz) frequency band. We have summarized all the trials of one subject because of different sample sizes for each trial. In each session, the data format for a single subject in the SEED dataset is 3394×62×5 ( sample×channel×frequency band ). However, what sets it apart from the SEED dataset is that trials for each session in the SEED-IV dataset are inconsistent, resulting in 851, 832, 822 samples in 3 sessions. The data format for a single subject in the SEED-IV dataset is 851/832/822×62×5 ( sample×channel×frequency band ).

### 4.3 Evaluation strategy

In this article, the strategy for model training includes both subject-dependent and subject-independent experiments.

In the subject-dependent experiment, we use ten-fold cross validation and leave-one-trial-out strategy to analyze each subject. For ten-fold cross validation strategy, the entire dataset is randomly divided into 10 equally sized subsets, each of which strives to maintain the overall distribution of the data. The model is trained using merged data from 9 subsets, and then evaluated on the reserved test set to obtain performance metrics such as accuracy. This process is repeated ten times to ensure that each subset has a chance to be used as a test set, resulting in 10 independent training and validation processes. Leave-one-trial-out strategy aims to evaluate the model’s generalization ability to new experiments. If there are N experiments of one subject, in each validation process, select one experiment as the test set and the remaining N-1 experiments as the training set. This process will be repeated N times, each time selecting a different experiment as the test set to ensure that each experiment has the opportunity to be used as data to validate the performance of the model.

In the subject-independent experiment, leave-one-subject-out cross validation strategy was adopted. This strategy is used to evaluate the model’s generalization ability to new individual data. Assuming there are N subjects, data from N-1 subjects is selected as the training set for each experiment, leaving one subject as the testing set until each subject’s data is tested once.

In terms of data label selection, we use the valence, arousal, dominance labels of the DEAP dataset, and the emotional state labels of the SEED and SEED-IV datasets.

### 4.4 Model training details

In the development of our DAMGCN model, it is necessary to quantify these model parameters in Dual Channel Attention Mechanism Block: Encoder, embedding vector, Multi-Head Attention. EEG data may contain more direct emotional signals compared to natural language processing tasks. The number of Encoders can start with fewer layers to avoid overfitting and maintain computational efficiency. The size of the embedding vector is determined based on the size and complexity of the dataset. In EEG emotion recognition tasks, it is possible to consider setting it between 32 and 64 because of the number electrode channels. The number of Multi-Head Attention can start with 4, which means it is possible to simultaneously focus on multiple aspects of the signal. For the Classifier Block, we use GELU activation function and two linear layers to gradually map high-dimensional features to the output dimension of emotion categories to increase the non-linear ability of the model. However, excessively large parameter settings may not result in significant performance improvements but could instead increase computational complexity. Based on experimental results and computational resources, we have made appropriate adjustments, with the parameter values shown in Table 2. Other parameters are adjusted through the first experiment of ten-fold cross validation. The number of epochs was set by early stopping strategy. After 200 epochs, the classification performance of the model did not show significant improvement and the training would be stopped. Batch size is conventionally established as a power of 2. Through our rigorous experimentation, it has been determined that a batch size of 64 facilitates more stable gradient descent. After experimenting with dropout rates between 0.1and 0.5, we selected 0.5 for optimal performance. We tested multiple learning rates from 1e-6 to 1e-1 and found that when the learning rate was 0.001, the model was able to perform better. The loss function and optimizer we used are Cross entropy and Adam. Our experimental platform relies on the hardware condition of NVIDIA GeForce RTX 3080 Ti and deep learning framework used was PyTorch 1.11.0. The parameter settings are shown in the Table 2.

**Table 2:** Parameters settings of DAMGCN.

Hyper-parameter of DAMGCN	Value or type
Number of Encoders	2
Size of the embedding vector	64
Number of multi-head attention	6
Linear layers in classifier block	2
Max number of epochs	200
Batch size	64
Dropout	0.5
Learning rate	0.001

### 4.5 Results and comparison

**Figure 4:**
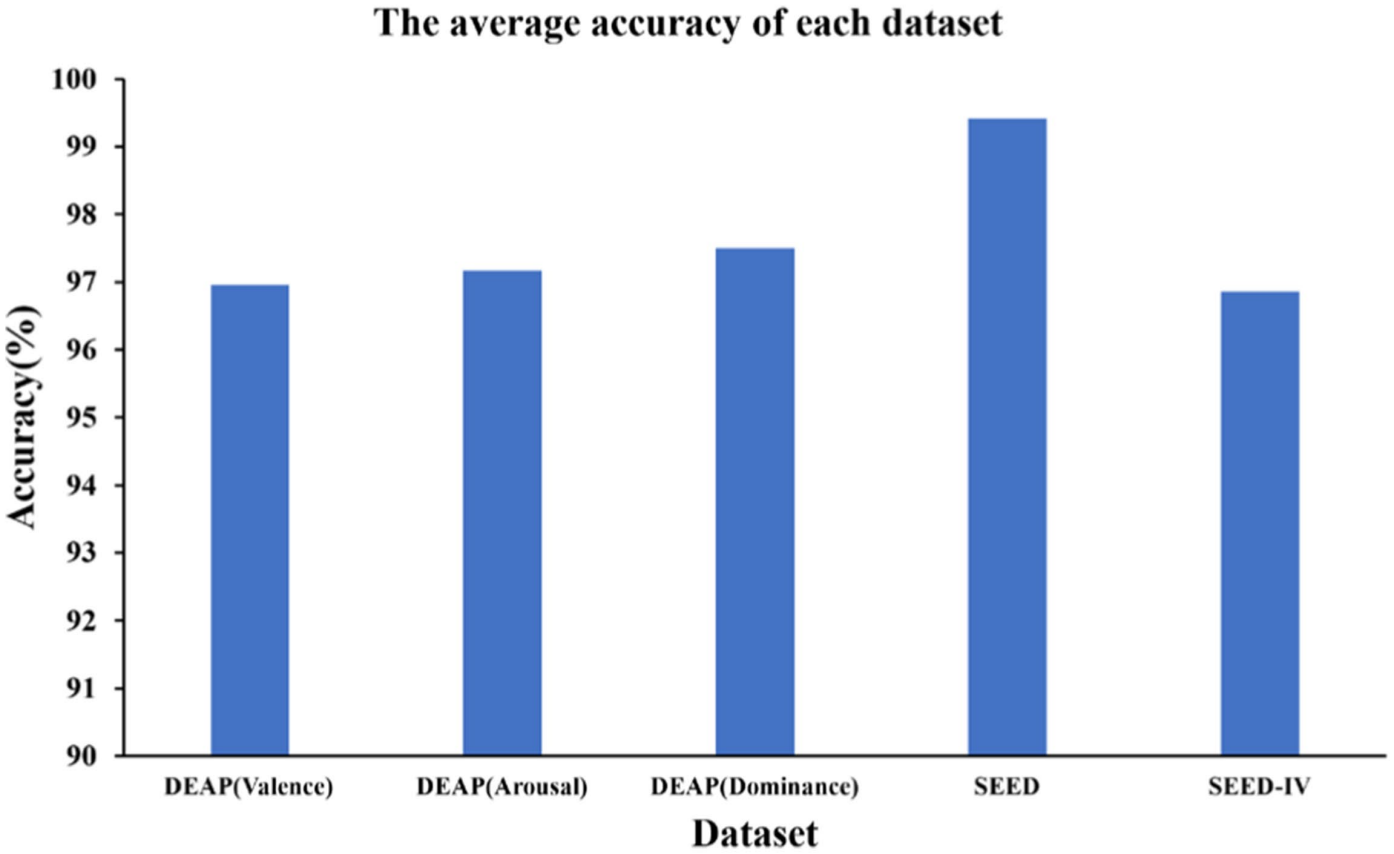
Average accuracy of DEAP, SEED and SEED-IV.

As shown in the Figure 4, we obtained ten-fold cross validation experiment’s average accuracies of 96.96, 97.17, 97.50% for the two-class dimension labels of valence, arousal, dominance in DEAP dataset. For the three-class labels in SEED dataset, we achieved an average accuracy of 99.42% in three sessions. And in the case of the four-class labels in the SEED-IV dataset, the average accuracy obtained was 96.86%. The data indicates that our model has achieved an accuracy of over 96% on various types of datasets, demonstrating its wide applicability across different datasets.

**Figure 5:**
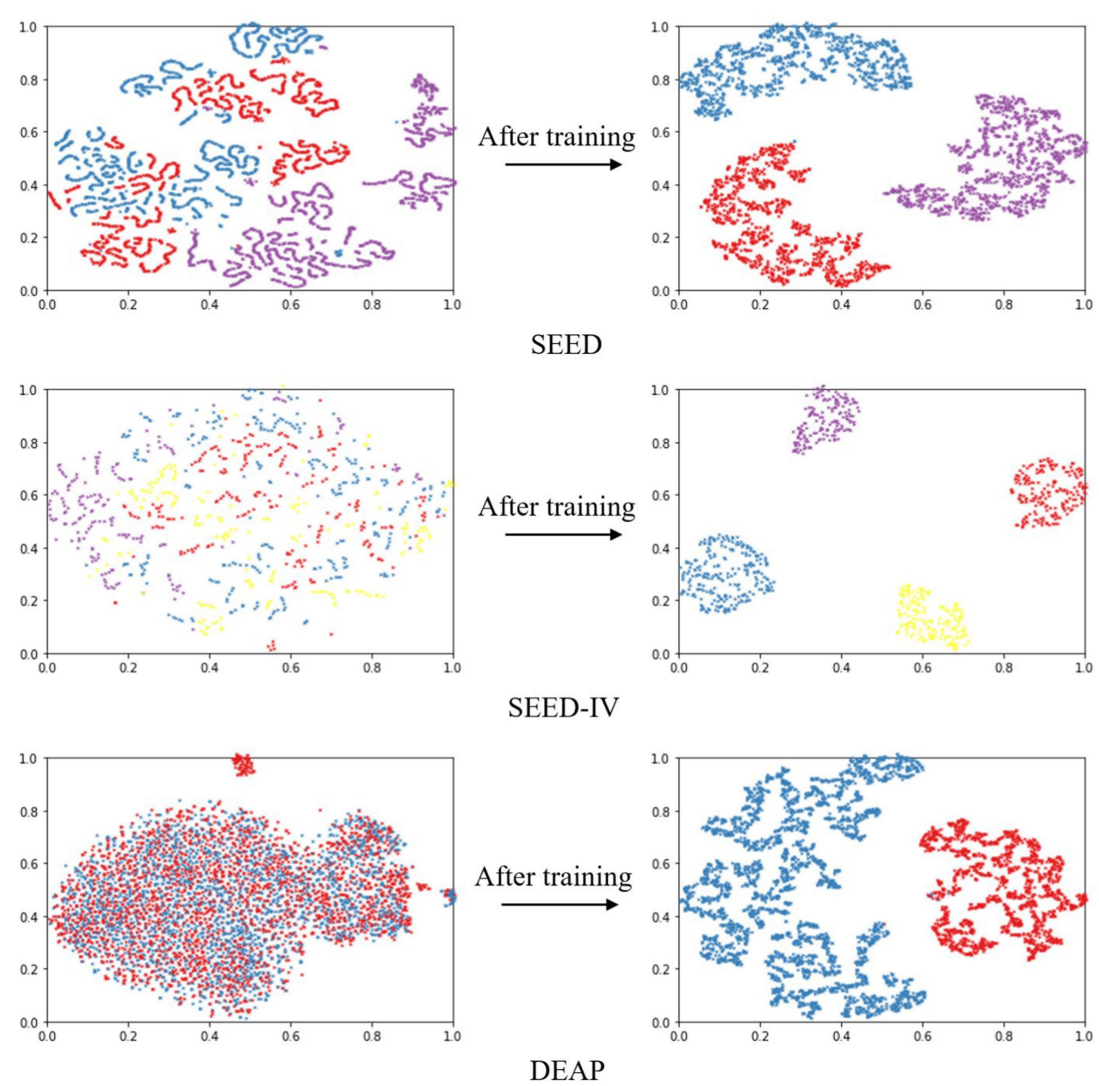
Visualization of 5% DE features of all subjects before and after training using DAMGCN.

**Figure 6:**
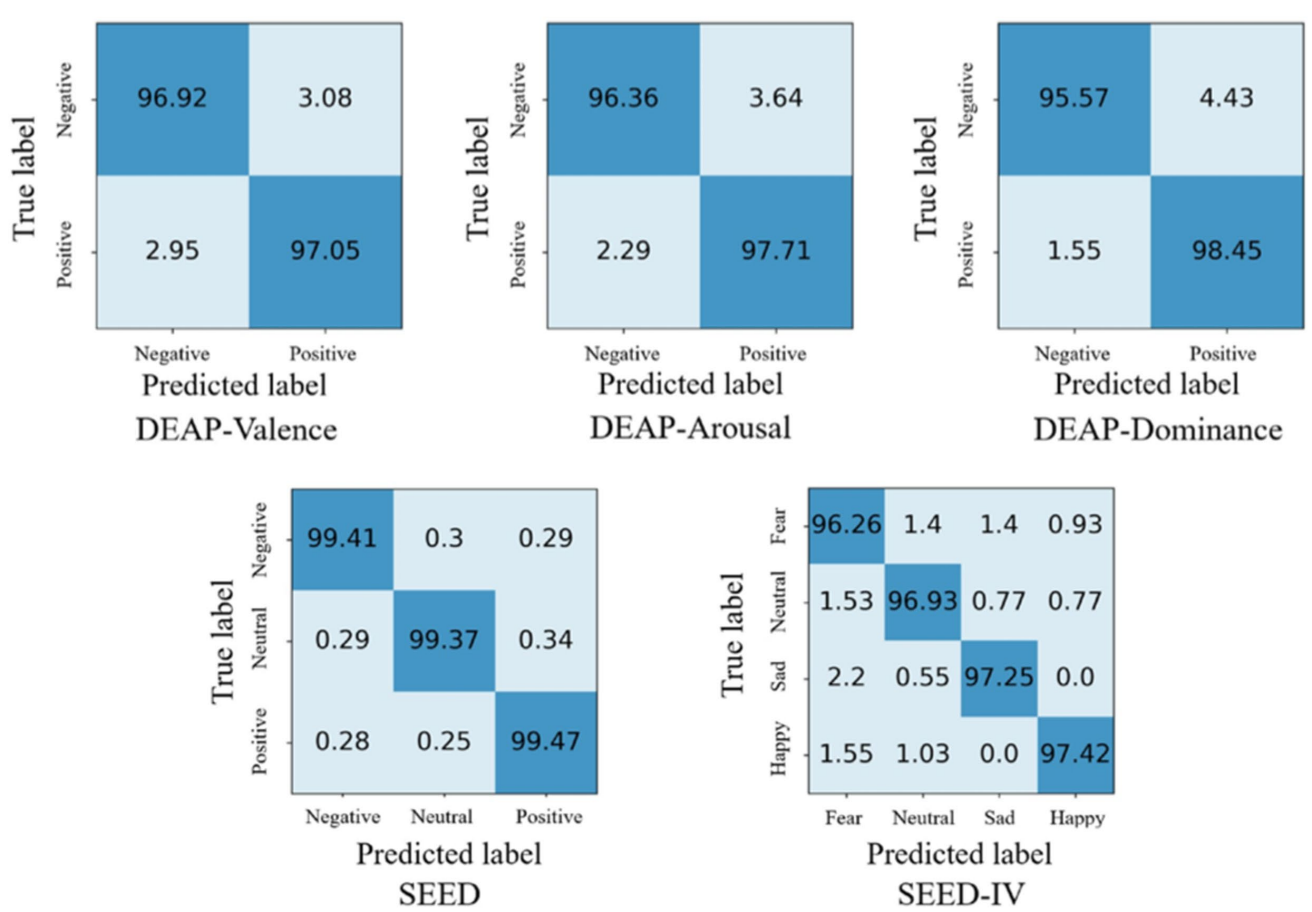
Confusion matrixes of the results on DEAP, SEED, SEED-IV.

Based on the results of ten-fold cross validation experiments, we selected 5% DE features of all subjects and plotted t-SNE in Figure 5. It can be observed that after DAMGCN training, the sample distribution becomes more distinct, and the level of disorder decreases. To analyze the performance of each dataset in different emotion categories more comprehensively, we have calculated confusion matrices using the proposed DAMGCN model in Figure 6. In the confusion matrix, the row sum represents the total number of samples, the diagonal elements represent the percentage of correctly classified samples for each emotion, and the remaining elements indicate the percentage of misclassified samples. Our findings reveal that the accuracy of classifying positive emotions consistently exceeds that of negative emotions. This suggests that the proposed method exhibits higher discriminative capability for positive emotions, which aligns with similar observations in other related works (Koelstra et al., 2012; Li et al., 2021; Guo et al., 2022).

To evaluate the performance of our method, we conducted comparative studies with relevant literature that employed the same experimental methods and datasets in Tables 3–5, which including traditional machine learning models as well as some state-of-the-art neural network models. Session-average represents the average accuracy across three sessions on SEED and SEED-IV datasets. Based on the comparison, it has been demonstrated that our proposed DAMGCN model outperforms existing algorithms in terms of accuracy and stability on the DEAP, SEED, and SEED-IV datasets. In addition, we extended our investigation through leave-one-trial-out strategy and subject-independent experiment, the outcomes delineated in Tables 6, 7 reveal that DAMGCN continues to exhibit comparative superiority in relation to existing methods. Comparing our results with existing graph neural network methods (Kipf and Welling, 2017), it can be concluded that DAMGCN designed with EEG signal characteristics performs better in emotion recognition tasks. Furthermore, Transformer’s attention mechanism can selectively focus on electrical signals in certain regions or frequency bands of EEG data that are more relevant to emotion recognition tasks, dynamically assigning weights to different features, thereby enhancing the influence of informative features while reducing less useful ones.

**Table 3:** The average accuracy /standard deviation (%) of different methods on DEAP two-class.

Method	Feature	Valence (acc/std)	Arousal (acc/std)	Dominance (acc/std)
SVM (Kumar and Nataraj, 2019)	PSD/DE	74/−	86/−	72/−
CNN-BiLSTM (Cui et al., 2022)	DE	94/−	−/−	−/−
CNN (Li et al., 2019)	DE	89.43/4.72	90.40/4.05	−/−
LSTM-RNN (Xing et al., 2019)	PSD	81.1/−	74.38/−	−/−
GCN (Kipf and Welling, 2017)	DE	82.56/2.11	80.47/3.84	85.23/2.32
MRGCN (Qiu et al., 2023)	DE	94.97/3.8	95.72/2.5	−/−
GLFANet (Liu et al., 2023)	DE	94.53/1.02	94.91/1.05	95.35/0.90
AP-CapsNet (Liu et al., 2023)	MobileNet	93.89/−	95.04/−	95.08/−
TR&CA (Peng et al., 2023)	Raw signal	95.18/2.46	95.58/2.28	95.78/2.16
ST-CLSM (Feng et al., 2022)	Raw signal	95.52/−	95.04/−	−/−
EESCN (Xu et al., 2024)	DE	94.56/4.18	94.81/3.62	94.73/4.12
DAMGCN (ours)	DE	96.96/2.24	97.17/2.35	97.50/2.03

**Table 4:** The average accuracy /standard deviation (%) of different methods on seed three-class.

Method	Feature	Session-average (acc/std)
CNN-BiLSTM (Cui et al., 2022)	DE	94.82/−
GCN (Kipf and Welling, 2017)	DE	92.54/1.89
MRGCN (Qiu et al., 2023)	DE	98.98/1.5
DPGAT (Li et al., 2021)	Raw signal	95.76/5.77
GLFANet (Liu et al., 2023)	DE	93.19/1.54
Bi-ViTNet (Lu et al., 2023)	PSD/DE	97.55/1.58
ACTNN (Gong et al., 2023)	DE	98.47/1.72
ST-CLSM (Feng et al., 2022)	Raw signal	96.20/−
DAMGCN (ours)	DE	99.42/0.24

**Table 5:** The average accuracy /standard deviation (%) of different methods on SEED-IV four-class.

Method	Feature	Session-average (acc/std)
SVM (Kumar and Nataraj, 2019)	PSD/DE	77.33/−
GCN (Kipf and Welling, 2017)	DE	82.86/3.64
Bi-ViTNet (Lu et al., 2023)	PSD/DE	88.08/6.32
ACTNN (Gong et al., 2023)	DE	91.90/5.43
ST-CLSM (Feng et al., 2022)	Raw signal	93.86/−
EESCN (Xu et al., 2024)	DE	79.65/8.22
DAMGCN (ours)	DE	96.86/1.33

**Table 6:** The average accuracy /standard deviation (%) of different methods (leave-one-trial-out strategy).

Method	Feature	DEAP (acc/std)	SEED (acc/std)	SEED-IV (acc/std)
SVM (Kumar and Nataraj, 2019)	PSD/DE	66.25/9.04	−/−	−/−
DBN-CRF (Chao and Liu, 2020)	Power features	76.13/−	83.46/−	−/−
GAT (Veličković et al., 2018)	DE	72.65/6.88	80.15/5.56	78.55/6.32
GCN (Kipf and Welling, 2017)	DE	75.86/4.35	83.56/2.98	81.05/4.73
DAMGCN (ours)	DE	78.21/5.64	90.4/2.61	83.55/3.88

**Table 7:** The average accuracy /standard deviation (%) of different methods (subject-independent).

Method	Feature	DEAP (acc/std)	SEED (acc/std)	SEED-IV (acc/std)
SVM (Kumar and Nataraj, 2019)	PSD/DE	59.6/−	56.73/16.29	51.78/12.85
VDM-DNN (Pandey and Seeja, 2022)	VMD	61.88/−	−/−	−/−
GCN (Kipf and Welling, 2017)	DE	58.4/13.56	62.4/10.14	59.13/14.55
LGG (Ding et al., 2022)	Raw signal	63.07/−	−/−	−/−
AP-CapsNet (Liu et al., 2023)	MobileNet	63.41/−	−/−	−/−
SSL-EEG (Xie et al., 2021)	Raw signal	−/−	67.52/12.73	53.62/8.47
MetaEmotionNet (Ning et al., 2024)	DE		77.5/0.088	61.2/0.083
A-LSTM (Li et al., 2022)	Raw signal	−/−	72.18/10.85	69.5/15.65
DAMGCN (ours)	DE	64.61/10.54	73.21/8.35	68.22/7.03

### 4.6 Interpretable analysis

**Figure 7:**
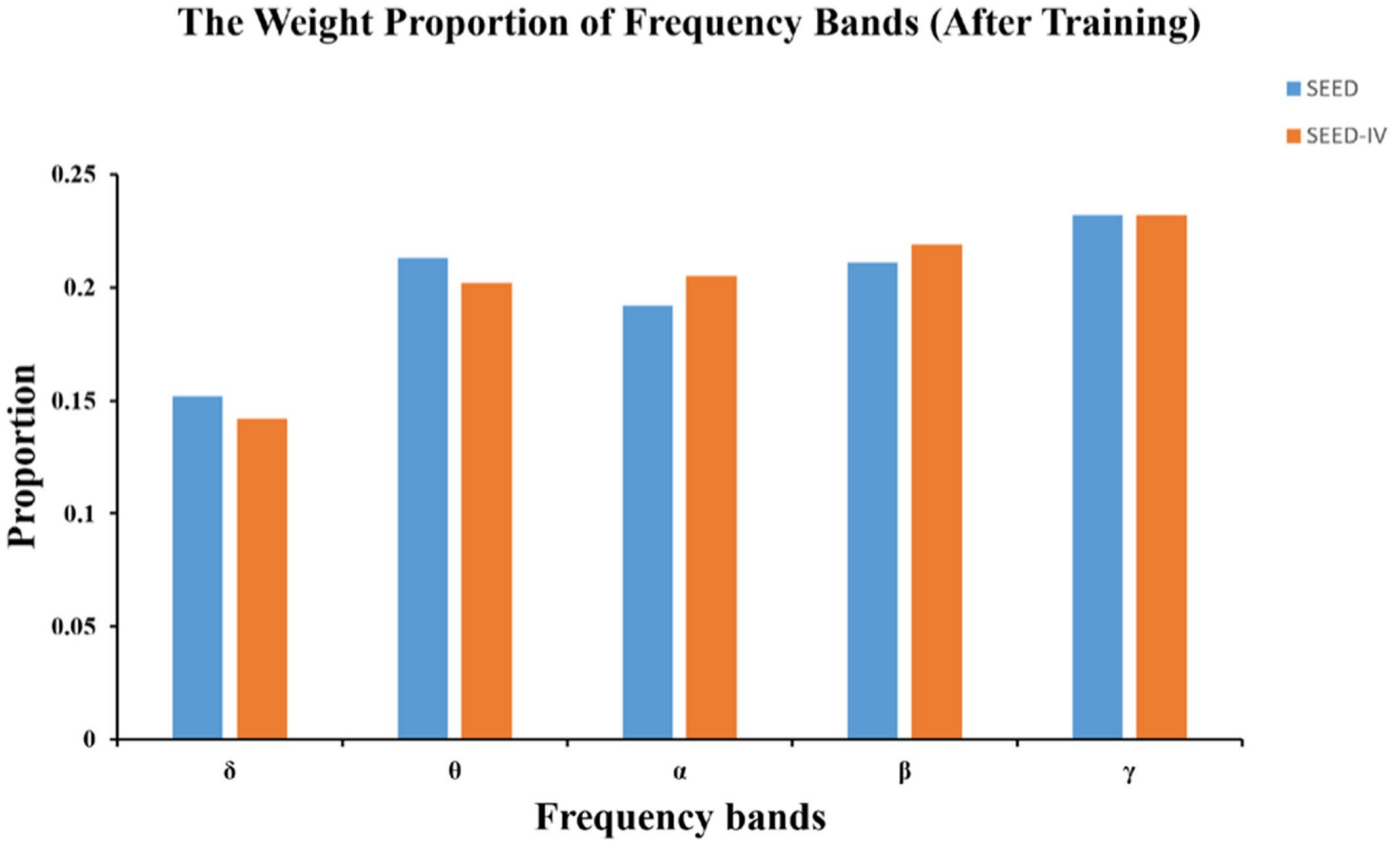
The weight proportion of frequency before and after training using DAMGCN.

Benefiting from the combination of the dual attention mechanism and GCN, our model has an advantage in interpretability. In frequency band analysis, the DEAP dataset is not included due to the absence of data in the α wave band (1–4 Hz). Figure 7 shows the parameter visualization after training convergence on the SEED and SEED-IV datasets, obtaining the contribution of different frequency bands to emotion recognition tasks.

The initial weight coefficients for each band before training are 0.2. After training, the weight δ coefficient of the band is the lowest and consistently below 0.2. This is similar to the conclusion from previous literature (Duan et al., 2013; Zhang et al., 2020), which indicate that the δ band is associated with unconscious states and often appears during deep dreamless sleep, while emotional responses typically occur during wakefulness, especially in γ frequency band that is more prominent. We speculate that the weight coefficients of δ should gradually approach 0 as the epochs increase. However, in Figure 7, the coefficient remains around 0.15, suggesting that during the optimization process, the model parameters might have stagnated at a local minimum or maximum, causing the model to become trapped in a local optimum. We attempted to increase the learning rate to mitigate this situation in 4.3 section. Unfortunately, a large learning rate destabilized the optimization process, causing the loss function of the model to gradually increase instead of decreasing, preventing the model from converging to a suitable solution and ultimately resulting in ineffective training results. The issue of local optima is inevitable in deep learning. As a result, it’s necessary to analyze from the trend of parameter changes rather than the results. This approach can provide us with directions for exploration in unknown domains and serve as a reliable way to validate conclusions drawn by previous researchers in clinical settings.

On the other hand, we extracted the attention matrix of the electrode channels and used degree centrality to evaluate the importance of nodes. The formula is as follows in Eq. (14):


(14) $$DC_{i}=\frac{k_{i}}{N-1}$$

N represents the number of nodes, $k_{i}=\overset{n}{\underset{j=1}{\sum }}d_{ij}$ represents the sum of the weights connected to current node and all other nodes. By calculating the mean of the degree centrality weights for all participants, we generated a distribution map of node importance in the brain regions involved in emotional activity.

**Figure 8:**
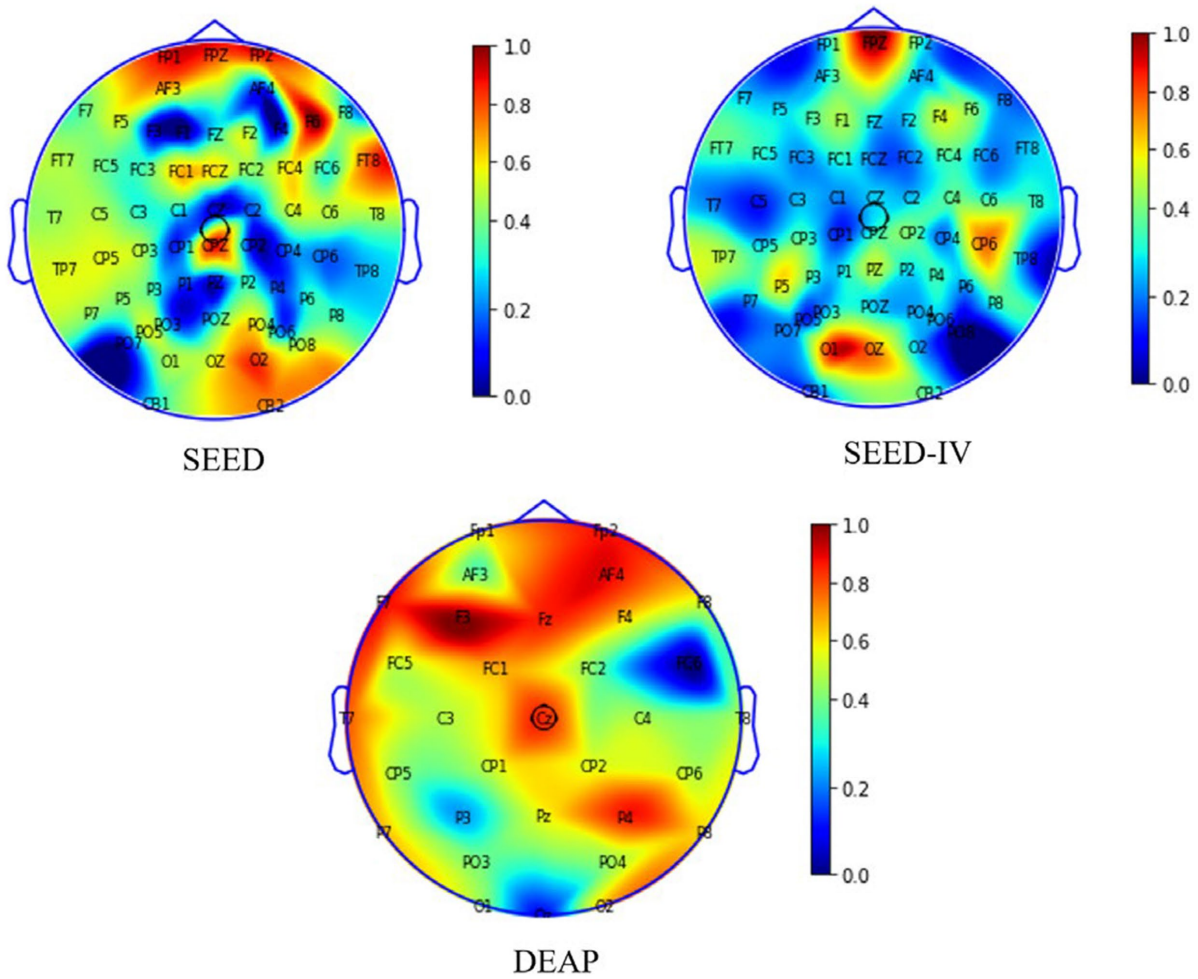
Visualization of brain regions weight distribution map on SEED, SEED-IV and DEAP.

The results in Figure 8 reveal that frontal lobe, temporal lobe, and occipital lobe regions exhibit higher node weight coefficients, indicating heightened emotional activity. Our finding aligns partially with the observations reported in reference (Liao et al., 2024 ; Nie et al., 2011; Li et al., 2024). In addition, we found that there is a greater difference in brain regions between the SEED and SEED-IV datasets through the comparison of the ab and c graphs. Based on the significant differences in the data in Tables 6, 7, it can be analyzed that as the number of electrode channels increases and the graph structure information becomes richer, the model can learn more common features, especially in subject-independent experiments. This can not only highlight the importance of graph convolutional neural networks but also offer insights for neuroscientists to assess the reliability of emotion recognition results based on brain activity regions.

### 4.7 Ablation

**Figure 9:**
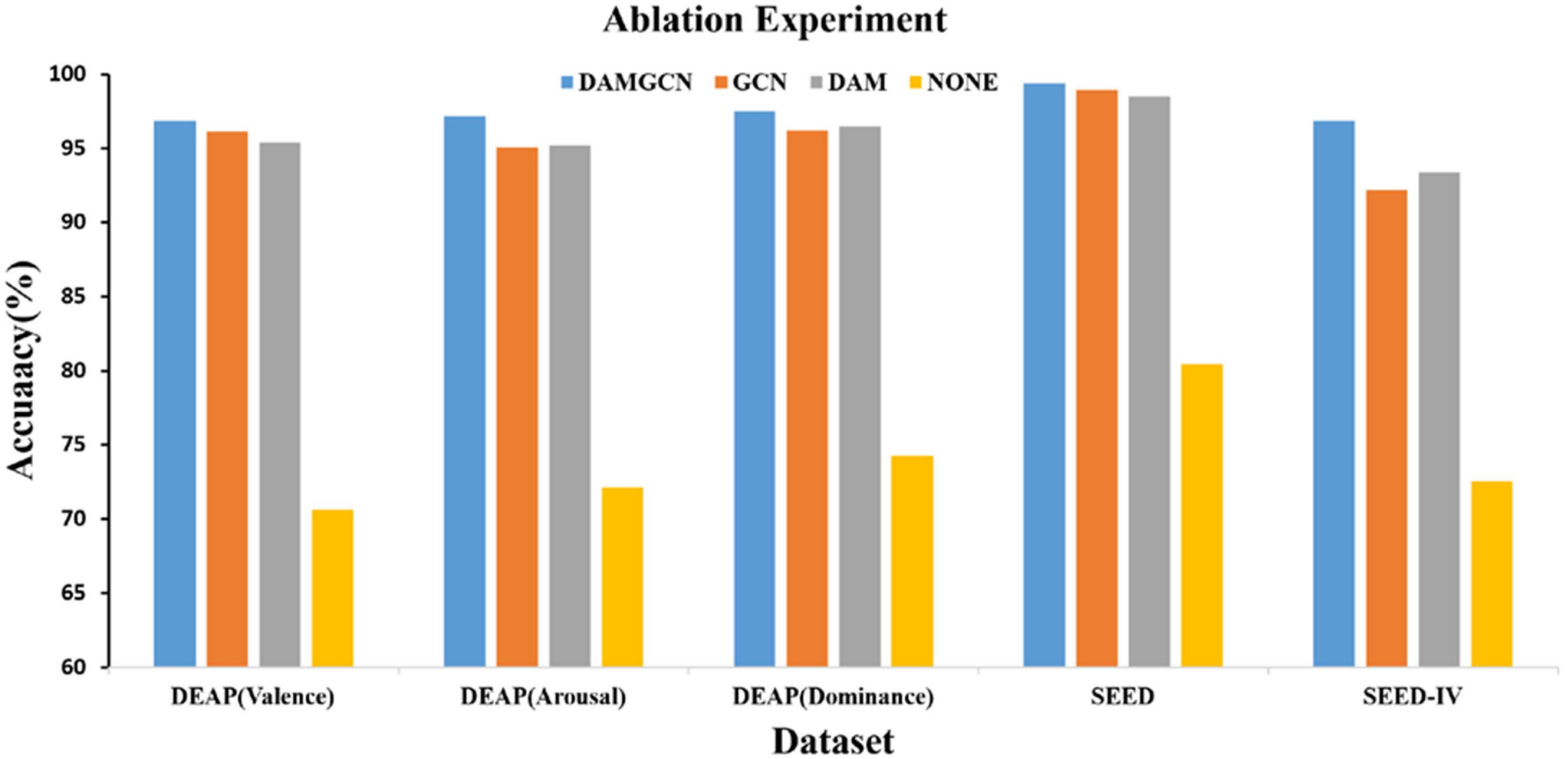
Accuracies of DAMGCN using different model structures.

We conducted subject-dependent ablation study by removing the GCN block and the DAM block to examine the importance of each block in our proposed model. The comparison of average accuracies of the DAMGCN, DAM, and GCN models on the DEAP, SEED, and SEED-IV datasets is shown in the Figure 9. When only the GCN block was involved in emotion recognition, the accuracy was 96.13, 95.1, 96.2, 98.98, 92.2%, resulting in a decrease of 0.73, 2.07, 1.3, 0.44, 4.66% in accuracy, respectively. This indicates that there might be channels unrelated to emotions among all electrode channels, and the GCN module was not effective in distinguishing them. DAM is the block proposed in this paper for effectively allocating channel weights. Without the GCN module, the accuracy on the DEAP dataset decreased by 1.46, 1.97, and 1% in the three different labels, while the accuracy on the SEED and SEED-IV datasets decreased by 0.92 and 3.46%. In addition, we removed the DAM and GCN modules, and compared the results obtained from only the linear layer model in Figure 9(labeled as None), it can be seen that the classification accuracy the accuracy was 70.62, 72.13, 74.26, 80.46, 72.54%. We can analyze the significant classification performance of DAM and GCN modules on EEG signals through data ablation experiments. We believe that the reason why the method proposed in this article performs well on these datasets can be attributed to the adaptability of the proposed model to EEG signals: EEG signals are electrical signals collected from multiple positions on the surface of the scalp, with a fixed spatial structure. The connections between different brain regions form a dynamic network, and different brain regions interact with each other through neural networks to influence emotional states. GCN naturally matches the spatial structure and dynamic network characteristics of EEG data through its ability to process graph structured data, enabling it to extract more complex and in-depth features from EEG signals. Transformer’s attention mechanism can selectively focus on electrical signals in certain regions or frequency bands of EEG data that are more relevant to emotion recognition tasks, dynamically assigning weights to different features, thereby enhancing the influence of informative features while reducing less useful ones.

In conclusion, our research results demonstrate the complementary roles played by the information aggregation of the Graph Convolutional Neural Network and the weight allocation of the Dual Attention Mechanism in extracting significant information from brain networks and ensuring stability in channel selection for classification.

## 5 Conclusion and future work

In this manuscript, we present the DAMGCN emotion recognition model, which combines the synergistic power of GCN and Transformer. The proposed model leverages the inherent connections between brain channels and utilizes the graph structure information to extract spatial topological features of the complex neural network. Additionally, it assigns weight coefficients to individual information to enable effective emotion classification. We conducted an extensive array of experiments on the DEAP, SEED, and SEED-IV datasets, and the results indicate that the model is competitive compared to state-of-the-art methods. Additionally, through ablative experiments, we corroborated the substantial contributions made by both the GCN block and the DAM block of our model in augmenting the classification performance. We also employed attention mechanism to visualize the significance of each EEG channel and different frequency bands in emotion recognition. Through this analysis, we observed that the weight coefficients associated with the δ frequency band were relatively low across most participants, suggesting a weak correlation between this particular EEG band and human emotions. Finally, our observations indicate a strong association between emotional activity and specific brain regions, notably the prefrontal and occipital lobes. This method we proposed offers a valuable framework for subsequent research endeavors in the field of emotion recognition.

In terms of models, our model requires more time to learn its parameters during the training phase, yet it remains susceptible to the challenge of getting trapped in local optima, a prevalent issue in many deep learning studies. Fortunately, we can mitigate this concern by focusing on the physical implications of parameter variations rather than solely relying on outcomes. In our subject-dependent and subject-independent experiments, the results of the subject-independent experiments were not very impressive. Therefore, our forthcoming study will attempt to introduce contrastive learning and transfer learning methods to improve the model, so that the model can learn common features between subjects to achieve high classification results in subject-independent experiments.

## Data availability statement

The original contributions presented in the study are included in the article/supplementary material, further inquiries can be directed to the corresponding author.

## Ethics statement

Ethical review and approval was not required for the study on human participants in accordance with the local legislation and institutional requirements. Written informed consent from the patients/ participants or patients/participants' legal guardian/next of kin was not required to participate in this study in accordance with the national legislation and the institutional requirements.

## Author contributions

WC: Conceptualization, Data curation, Investigation, Methodology, Project administration, Resources, Software, Validation, Visualization, Writing – original draft, Writing – review & editing. YL: Data curation, Formal analysis, Software, Supervision, Validation, Visualization, Writing – review & editing. RD: Data curation, Formal analysis, Funding acquisition, Supervision, Validation, Writing – review & editing. YD: Investigation, Project administration, Resources, Software, Writing – review & editing. LH: Funding acquisition, Project administration, Supervision, Validation, Writing – review & editing.
